# Evaluation of the Learning Environment for Diploma in Family Medicine with the Dundee Ready Education Environment (DREEM) Inventory

**DOI:** 10.3352/jeehp.2010.7.2

**Published:** 2010-11-29

**Authors:** A. Sattar Khan, Zekeriya Akturk, Tarek Al-Megbil

**Affiliations:** Centre for Postgraduate Studies in Family Medicine, Riyadh, Saudi Arabia.

**Keywords:** Dundee Ready Education Environment, Postgraduate Studies, Family Medicine, Teaching Environment

## Abstract

The primary healthcare system is at a turning point in Saudi Arabia. However, the sustainability of family medicine as the core element of that system is increasingly being called into question because of lack of family physicians. In keeping view this problem; a postgraduate diploma program in family medicine has started in 2008. A validated measure of educational environment i.e., Dundee Ready Education Environment (DREEM) questionnaire consisting of 50 questions having five domains of perception was administered to all 13 trainees of the diploma course at the completion of the program to check their perception about learning evironment. The trainees comprised of 4 males (40%) and 6 females (60%). The overall score showed more positive than negative side (147/200). There is no significant difference (*P* > 0.05) in the mean scores of five different domains of perception. The subclasses of five domains showed that teaching perceived as positive by 50%, moving towards right direction by 80%, feeling more positive by 50%, positive attitude by 80% and the 70% scored the course as not too bad. The overall high score and positive attitude towards the course assures the better teaching environment. However, there are areas to improve and it requires continuous evaluation.

The learning environment has its own impact on behavior of learning and a good environment enhances the ability of learning. The importance is recognized by learners and teachers. Students' perception of the environment within which they study has been shown to have a significant impact on their behavior, academic progress and sense of well-being [1]. Nevertheless different methodologies have been applied to investigate educational environment. Among them, the recent one includes qualitative approaches [2] or the use of questionnaires [3]. However, of these, only the Dundee Ready Educational Environment Measure (DREEM) questionnaire [4] is more focused to the environment experienced by students on medical and healthcare-related courses. This tool was build up by an international Delphi panel, and has been applied to a number of undergraduate courses for health professionals worldwide. The inventory was validated by over 1,000 students world-wide and is now being used widely in order to measure and 'diagnose' undergraduate educational climates in the health professions, having been translated into Spanish, Portuguese, Arabic, Swedish, Norwegian, Malay and Thai and used in several settings including the Middle East [5]. It is currently being used in the UK, Canada, Ireland, Thailand, Indonesia, Malaysia, Norway, Sweden, Venezuela, Brazil, the West Indies, Sri Lanka, Oman and the Yemen.

The Ministry of Health in Saudi Arabia at centre for postgraduate studies in Family Medicine has started first time a diploma program in family medicine. It is a comprehensive 14-month's program covering all aspects of family medicine. Initially 13-students have been enrolled and completed their studies in year 2009. In this cross sectional pilot study, all students were asked to fill the DREEM inventory; however three did not submit their questionnaires. The DREEM inventory questionnaire includes of 50 items [5], each scored 0-4 on a 5-point scale. The statements which are negative considered in reverse order, so that high scores on these items indicate disagreement will become a positive result. This questionnaire produces an overall "score" for the course and results were analyzed with Chi Square and the Mann-Whitney U test. The overall statements may also be subdivided to provide different student perceptions in the groups of 5 different aspects [6] of the educational environment:

- Perceptions of learning
- Perceptions of teachers
- Academic self-perception
- Perceptions of atmosphere
- Social self-perceptions

The results revealed that median age of the male participants was 25.50 years (4 males) and female participants was 33 years (6 females). The perception analysis shows that 50% students perceived teaching as negative whereas 80% agreed that they are moving in a right direction (Table 1). Academic self perception shows more satisfaction as 50% feeling more on positive side. The overall perception was good. Not a single perception was scored below the 2 mean values. The overall median DREEM score was 118.5/200 (59.25%) (Table 2). There is no significant difference (*P* > 0.05) in the mean scores of five different domains of perception. The Table 3 highlights the rating of the students responses on 50-items individually.

So far this is the first study to report results from the complete postgraduate cohort of a diploma program for family medicine. The DREEM questionnaire has provided an overview of student opinion throughout the study period (14-months) and allowed areas of concern to be highlighted. Analysis of the identified concerns will form the basis of strategic planning in order to institute remedial action, and on which to focus institutional resources. The study shows that the perception of teachers is better than the other dimensions. One reason for this might be that the diploma program in our setting is rather new and hence, the facilities are not settled yet. However, a study at the well-settled college of medicine in King Saud University found a similar result [7], which could indicate that there are other factors beyond facilities. The overall median score being at around 60% is another indication that there is some area to improve. A study in West Mislands revealed mean DREEM scores of 70% of the total [8]. DREEM global score of a Nigerian medical school was reported as 118/200 and that of a Nepalese health professions institution was reported as 130/200 [9]. DREEM global score of the faculty of medical sciences in Trinidad was reported as 109.9 [10]. Mayya and Roff [11] reported global scores of 107.44/200 in an Indian medical school and Varma [7] reported scores of 139/200 in undergraduate schools. When compared with these figures our results demonstrate that the SDFM evaluations ranks somewhere around the average in the literature. Item-wise ratings of the two questions suggest that most aspects of the climate could be enhanced. Students in general are more critical about the quality of teaching, especially in the areas of student participation in the class, provision of clear learning objectives, course being boring, trainee centred teaching, and keeping attached to the timetable. Learning atmosphere on the other hand received higher scores. Mayya's study revealed that students in general are more critical about the quality of teaching and the general climate of the school [10]. Continuous quality improvement and innovation are very essential in a medical school. Most researchers agree upon the usefullness of the DREEM inventory as a useful basis for strategic planning and resource utilization [7, 12]. Nevertheless, due to small sample size, the generalization of results might not be good idea however, it gives some clues or hints about the program and need to follow up for coming years. Further research on the use of DREEM inventory, comparing different diploma programs with follow up surveys, may be useful for educators to ensure and maintain high quality educational environments.

## Figures and Tables

**Table 1:**
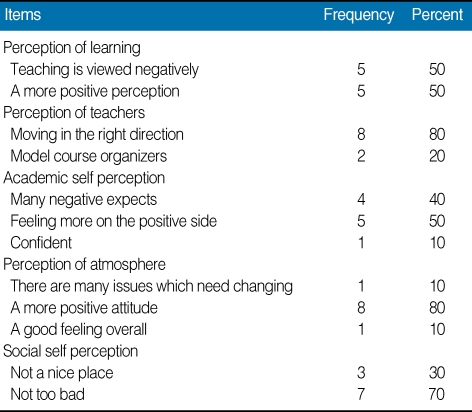
Perception of students

**Table 2:**
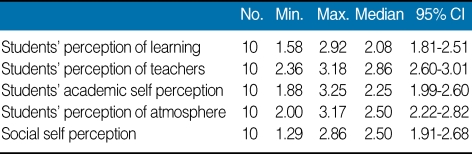
Median scores of different perceptions

**Table 3:**
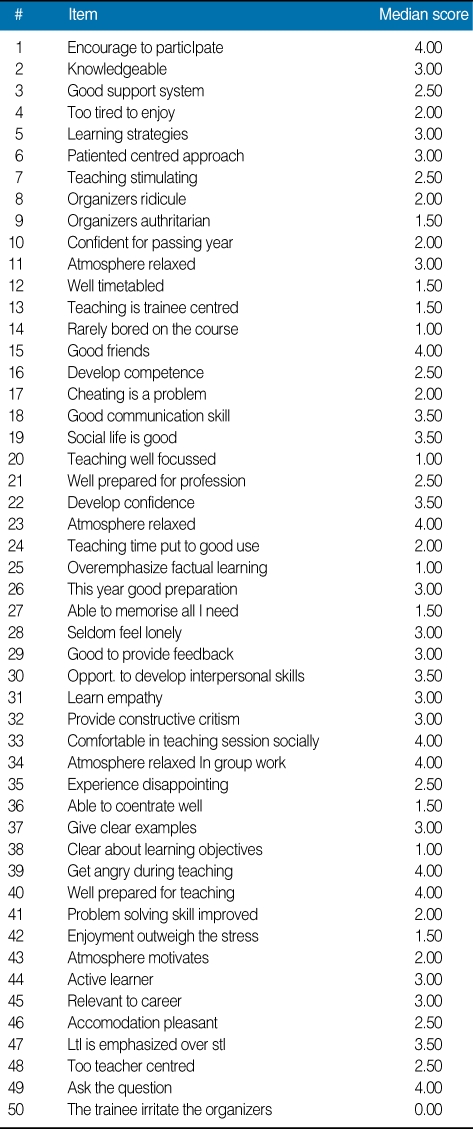
Item-wise ratings of the Dundee Ready Educational Environment Measure (DREEM) questions
